# Neuronal sub‐compartmentalization: a strategy to optimize neuronal function

**DOI:** 10.1111/brv.12487

**Published:** 2019-01-04

**Authors:** Alessandra Donato, Konstantinos Kagias, Yun Zhang, Massimo A. Hilliard

**Affiliations:** ^1^ Clem Jones Centre for Dementia Research Queensland Brain Institute, The University of Queensland Brisbane Queensland Australia; ^2^ Department of Organismic and Evolutionary Biology Center for Brain Science, Harvard University Cambridge MA 02138 U.S.A.

**Keywords:** unipolar neurons, polarization, compartmentalization, patterning, neuronal development, neuronal activity, axonal and dendritic sub‐compartments

## Abstract

Neurons are highly polarized cells that consist of three main structural and functional domains: a cell body or soma, an axon, and dendrites. These domains contain smaller compartments with essential roles for proper neuronal function, such as the axonal presynaptic boutons and the dendritic postsynaptic spines. The structure and function of these compartments have now been characterized in great detail. Intriguingly, however, in the last decade additional levels of compartmentalization within the axon and the dendrites have been identified, revealing that these structures are much more complex than previously thought. Herein we examine several types of structural and functional sub‐compartmentalization found in neurons of both vertebrates and invertebrates. For example, in mammalian neurons the axonal initial segment functions as a sub‐compartment to initiate the action potential, to select molecules passing into the axon, and to maintain neuronal polarization. Moreover, work in Drosophila melanogaster has shown that two distinct axonal guidance receptors are precisely clustered in adjacent segments of the commissural axons both *in vivo* and *in vitro*, suggesting a cell‐intrinsic mechanism underlying the compartmentalized receptor localization. In Caenorhabditis elegans, a subset of interneurons exhibits calcium dynamics that are localized to specific sections of the axon and control the gait of navigation, demonstrating a regulatory role of compartmentalized neuronal activity in behaviour. These findings have led to a number of new questions, which are important for our understanding of neuronal development and function. How are these sub‐compartments established and maintained? What molecular machinery and cellular events are involved? What is their functional significance for the neuron? Here, we reflect on these and other key questions that remain to be addressed in this expanding field of biology.

## I. INTRODUCTION: SUB‐COMPARTMENTS IN NEURONS

**Figure 1:**
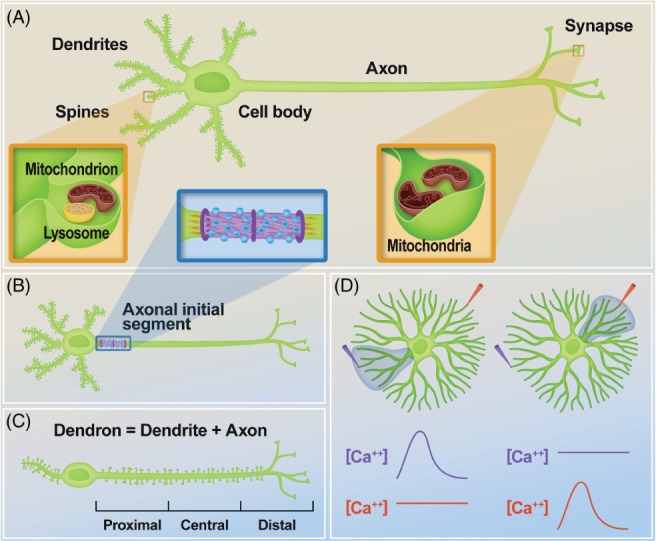
Neurons contain compartments, which can be further divided into sub‐compartments. (A) Neurons are polarized cells with three main compartments: dendrites, cell body (or soma), and axon. Within each of these compartments, there are domains (sub‐compartments) that can be defined based on their morphology and/or function, such as the synaptic spines present in dendrites, and the presynaptic boutons present in axon terminals. Both are crucial for synaptic activity and plasticity. Organelles can localize in these discrete sub‐compartments to acquire specific functions in spine formation and synaptic transmission; (B) Through immunohistochemistry and electrophysiology it is possible to identify the axonal initial segment; this proximal section of the axon closest to the soma contains an increased number of sodium channels that are necessary for the initiation of the action potential, and a cytoskeletal architecture that functions as a filter for cytoplasmic transport to the distal axon; (C) The neurite of the gonadotropin‐releasing hormone (GnRH) neurons is referred to as a ‘dendron’ because it functions as both a dendrite and an axon. This long projection is divided into three functional sub‐compartments: the proximal dendron, which is necessary for action potential initiation and conduction, the central dendron, which is implicated in action potential conduction, and the distal dendron, which is required for integration of sub‐threshold stimuli. It is still not known how pre‐ and postsynaptic elements are precisely organized in this configuration; (D) The dendrites of starburst amacrine cells, which are involved in detecting the direction of the movement of visual stimuli, show calcium compartmentalization. Schematic representation of the calcium recordings from two opposite regions of the dendritic arborizations (orange and purple cones) during light illumination (blue shaded area), showing an increase in the calcium concentration [Ca^++^] in the illuminated dendrites but not in dendrites on the opposite side. Adapted from Euler *et al*. (2002).

Neurons are specialized cells with a high level of polarization defined by the presence of three major compartments: the dendrites, a cell body (or soma) and an axon (Fig. 1A). Dendrites are specialized to receive electrochemical signals, which are then processed and transferred through the cell body and along the axon to be transmitted to the target cell/s. However, this broad definition of neurons containing just three major compartments is simplistic for two reasons. First, it does not consider the unipolar neurons in which the unique neurite has a mixed axon/dendrite identity, and second, it does not take into account those functional and molecular sub‐compartments that have been identified and characterized by a growing body of studies (Fig. 1).

Some clear and well‐studied sub‐compartments within dendrites and axons, such as the dendritic spines and the presynaptic boutons, were recognized decades ago, in part due to their distinct morphology. Dendritic spines were first observed as protrusions on the dendrites through gold staining by Ramon y Cajal, and represent the dendritic sub‐compartments that receive inputs from a single axon (Gray, 1959; Guillery, 2000). More advanced labelling and imaging techniques revealed that the structure and function of spines are dynamically regulated by neuronal activity, play a critical role in excitatory synaptic signalling, and are further divided into functional microdomains (reviewed in Berry & Nedivi, 2017 and Chen & Sabatini, 2012). Similarly, a large body of work through both electron and confocal microscopy has revealed the complex and specialized morphology of synaptic boutons designed to pass information to postsynaptic cells (Palay, 1956; Sabo, Gomes, & McAllister, 2006; Vulovic, Divac, & Jakovcevski, 2018). The differentiation of presynaptic terminals has been reviewed recently, with emphasis on the mechanisms underlying the delivery and recruitment of presynaptic molecules to axonal regions that eventually give rise to boutons, as well as the final differentiation that depends on retrograde signals from postsynaptic neurons (Pinto & Almeida, 2016).

In this review, we highlight reports of functional and molecular sub‐compartmentalization, also referred to as intra‐neurite patterning, within the major neuronal compartments that do not have distinct morphology. For example, both *in vitro* and *in vivo* models provide evidence of neurites with shared axonal and dendritic properties, axonal compartmentalization of membrane proteins, such as axon guidance receptors and ion channels, and clustering of intracellular components within neurites, which include protein aggregates, organelles and ions (Fig. 1). These findings raise intriguing questions on the development, maintenance, and function of these sub‐compartments (Katsuki *et al.,* 2011).

## II. SUB‐COMPARTMENTALIZATION IN NEURITES WITH SHARED AXONAL AND DENDRITIC PROPERTIES

**Figure 2:**
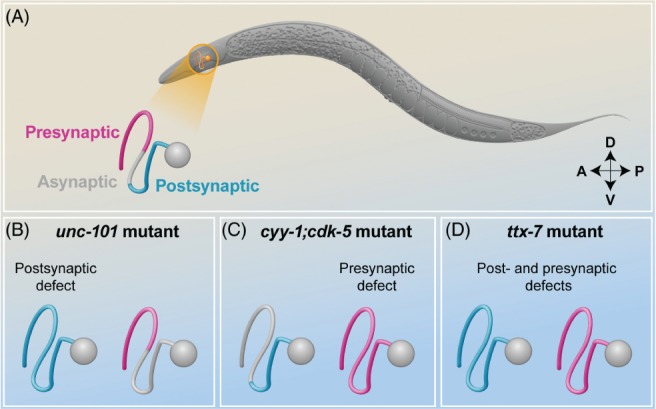
Compartmentalization of synaptic molecules in the Ring interneurons A (RIAs). (A) Schematic representation of Caenorhabditis elegans in lateral view; the head is on the left, the tail is on the right, the dorsal side is up and the ventral side is down. The RIA interneurons are located in the head of the animal (orange) and the left RIA is schematically represented. The diagram of the wild‐type RIA neuron shows the synaptic compartmentalization of the RIA neurite, with the exclusively postsynaptic proximal region (blue), the asynaptic isthmus region (grey), and the distal region mixed with presynaptic and postsynaptic sites (magenta); (B) In *unc-101* mutant animals the postsynaptic region, defined by the localization of the glutamate receptor GLR‐1 (blue), extends to the entire length of the neurite as a result of the lack of receptor retrieval from the distal region, whereas the distal presynaptic region (magenta), defined by the localization of the Ras‐associated binding protein RAB‐3, is unaltered; (C) Conversely, in double mutant *cyy-1;cdk-5* animals the presynaptic molecules diffuse towards the dendritic compartment in a dynein‐dependent manner, leaving the distribution of postsynaptic receptors unchanged; (D) Finally, in *ttx-7* mutant animals, both the distal presynaptic regions and postsynaptic regions are altered and redistributed throughout the neurite.

Although many neurons have presynaptic and postsynaptic sites that are physically separated in axons and dendrites, certain unipolar neurons contain these elements within the same neurite, thereby conferring a mixed axonal and dendritic identity. How pre‐ and postsynaptic sites are formed and organized in the same neurite is still unknown. In *Caenorhabditis elegans*, the bilateral head Ring Interneurons A (RIAs), which play critical roles in thermotaxis, navigation and learning (Mori & Ohshima, 1995; Ha *et al.,* 2010; Jin, Pokala, & Bargmann, 2016; Liu *et al.,* 2018), have a single unbranched neurite. The RIA neurite can be divided into a proximal region that is postsynaptic and a distal region that contains both postsynaptic and presynaptic sites; these two regions are separated by a section that appears void of any pre‐ or postsynaptic markers, referred to as the asynaptic section (Fig. 2A). The expression of some postsynaptic molecules, such as the glutamate receptor GLR‐1, is restricted to the proximal region, whereas common presynaptic markers, such as the small GTPase Ras‐associated binding protein RAB‐3 and the synaptobrevin SNB‐1, can be found in the distal region (Fig. 2A) (Tanizawa *et al.,* 2006; Margeta, Wang, & Shen, 2009). How this structure is generated has just started to be elucidated. The subcellular distribution of the synaptic proteins in the RIA neurite requires the medium subunit of the clathrin adaptor AP‐1 complex UNC‐101 (uncoordinated), the lithium‐sensitive enzyme abnormal ThermoTaXis/Inositol MonoPhosphatase TTX‐7/IMPA1, which converts inositol monophosphates to inositol, and two cyclin‐dependent kinase (CDK) pathways regulated by the cyclin y homologue CYY‐1 and the cyclin‐dependent kinase CDK‐5 (Tanizawa *et al.,* 2006; Margeta *et al.,* 2009; Ou *et al.,* 2010). Mutating *unc‐101* expands the localization of several postsynaptic receptors in the RIA neurite, including GLR‐1, from the proximal/dendritic region to the distal region in a cell‐autonomous fashion (Margeta *et al.,* 2009) (Fig. 2B). In this context, UNC‐101 enriches postsynaptic receptors in the proximal region of the RIA neurite by removing them from the distal region, and preventing the lateral diffusion of membrane proteins (Margeta *et al.,* 2009).

By contrast, mutating *cyy‐1* and *cdk‐5* results in a redistribution of presynaptic markers throughout the entire length of the RIA neurite (Margeta *et al.,* 2009; Ou *et al.,* 2010) (Fig. 2C). Ou *et al*. (2010) proposed that the CDK pathways regulate the distribution of presynaptic molecules by inhibiting dynein‐dependent retrograde transport; thus, when the CDK pathways are inactivated, the presynaptic molecules are mis‐localized to dendrites in a dynein‐dependent manner. These findings provide additional mechanisms whereby axonal trafficking is regulated. Finally, mutating TTX‐7 disrupts the localization of both post‐ and presynaptic molecules in the RIA neurite (Fig. 2D), resulting in defective thermotaxis (a navigational behaviour regulated by RIA neurons under certain experimental conditions), demonstrating that the compartmentalization of synaptic molecules is of major functional importance (Tanizawa *et al.,* 2006).

In *Drosophila melanogaster,* a number of sensory and motor neurons are also unipolar (Rolls, 2011) with the main neurite shaft containing both dendritic and axonal structures. In the central nervous system of the fruit fly, the sole primary neurite extends from the cell body within the cortex towards the neuropil that is void of somata. The neurites in the neuropil generate an elaborate arborization that contains both pre‐ and postsynaptic sites; output synapses, labelled by presynaptic proteins, such as synaptobrevin and synapsin, are restricted to the medial longitudinal fascicles, whereas the side branches from the transverse primary neurite are likely postsynaptic (Lohr *et al.,* 2002). However, the mechanisms that regulate synaptic compartmentalization in these neurons remain unknown.

In mammals, the gonadotropin‐releasing hormone (GnRH) neurons, which control fertility, are bipolar, with a long dendrite that extends for about 1000 μm. For many years, scientists struggled to identify the axonal structure of these neurons until it was revealed that the process of the GnRH neurons functions as both an axon and a dendrite (Fig. 1C) (Herde *et al.,* 2013). Interestingly, this neurite has a site which is required for spike initiation, conducts action potentials and has dendritic spines along its entire structure, and has therefore been renamed a ‘dendron’ (Herde *et al.,* 2013). These properties suggest that the dendron must have a unique way of integrating information. The proposed model postulates that the dendron contains three compartments with different computational functions (Iremonger & Herbison, 2015). The proximal dendron, which extends up to 500 μm from the cell body, regulates the initiation and conduction of action potentials. The central dendron conducts the action potential initiated in the proximal dendron; yet, the role of synaptic innervation in this region is not clear, as the synaptic inputs are too far away from the spike initiation site to cause excitability. The distal dendron, which integrates stimuli that are below the spiking threshold, controls GnRH release in a calcium‐dependent and action potential‐independent manner (Iremonger & Herbison, 2015). The authors proposed that this particular configuration allows the GnRH neurons to optimize GnRH secretion, which would not be possible if the dendrites were separated from the axon.

Thus, our knowledge of how axonal and dendritic domains are patterned in unipolar neurons is still preliminary, and it remains puzzling how pre‐ and postsynaptic molecules are organized in neurons with mixed dendritic and axonal identities.

## III. AXONAL AND DENDRITIC COMPARTMENTALIZATION

The axon and the dendrites of a neuron are not uniform entities and often contain domains characterized by specific membrane and cytosolic composition. One widely studied example is the axonal initial segment (AIS) (Palay *et al.,* 1968), which plays a critical role in initiating action potentials (Nelson & Jenkins, 2017) (Fig. 1B). Previous studies using immunohistochemical and electrophysiological approaches identified a high density of ion channels, including voltage‐gated sodium channels, in the AIS membrane, revealing the mechanisms whereby the AIS mediates the initiation of action potentials (Kole *et al.,* 2008). The distinct membrane properties of the AIS domain are maintained in part by a diffusion barrier that blocks the lateral diffusion of proteins and lipids between the AIS and the adjacent membrane regions (Winckler, Forscher, & Mellman, 1999; Nakada *et al.,* 2003). The AIS is also characterized by a distinct cytoplasmic composition that acts as a filter to block the transport of selected molecules between axonal and somatic compartments (Song *et al.,* 2009). Importantly, disruptions of the AIS have been implicated in pathologies of the nervous system and in brain injuries. Changes in the membrane composition or the cytoskeletal organization of the AIS in neurons of the central nervous system have been observed in a mouse model of epilepsy or following neuronal injuries (Schafer *et al.,* 2009; Wimmer *et al.,* 2010). A recent study in cultured hippocampal neurons found that hyperphosphorylated tau, a molecular correlate of neuronal degeneration in Alzheimer's disease, shifts the AIS to a more distal region of the axon, thereby reducing neuronal excitability (Hatch *et al.,* 2017). Together, these examples highlight the functional importance of the AIS compartment in neuronal activity under normal and disease conditions.

Myelinated axons of mammalian neurons have an additional type of sub‐compartmentalization that is represented by the nodes of Ranvier. These are localized in the regions void of myelin and contain clusters of sodium channels, cell adhesion molecules and scaffolding proteins and are required for the efficient propagation of action potentials (Waxman & Ritchie, 1993). The formation and maintenance of AIS and nodes of Ranvier require intrinsic and extrinsic mechanisms, which have been reviewed recently (Normand & Rasband, 2015).

**Figure 3:**
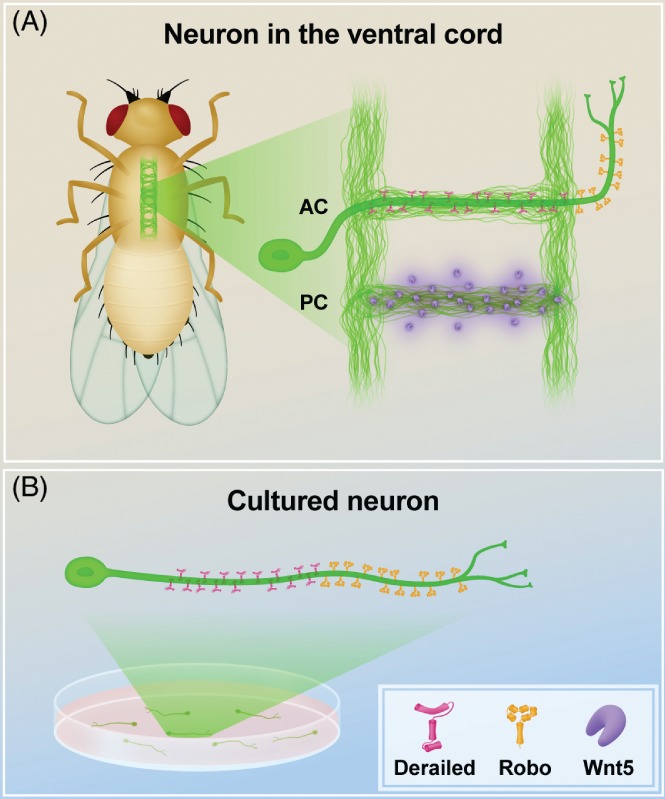
Axon guidance receptors compartmentalize in the axon of Drosophila melanogaster neurons. (A) In D. melanogaster, the axons of commissural neurons cross the midline of the ventral nerve cord through the anterior commissure (AC), avoiding the repellent Wingless/Integrated Wnt5 (purple) present on the posterior commissure (PC), and then extend anteriorly. These neurons display striking axonal compartmentalization of the axon guidance receptor molecules Derailed (pink), localized to the proximal region, and Roundabout (ROBO) molecules (orange), localized to the distal region; (B) Intrinsic mechanisms are required for this compartmentalization, as the same pattern of localization is also observed in the neurons in culture in the absence of external guidance cues.

Axonal compartmentalization of membrane proteins also plays a critical role in regulating neurodevelopment, particularly during axon outgrowth and guidance. This well‐studied process is regulated by extracellular guidance cues that induce cytoskeleton changes in growth cones to direct neurite extensions (reviewed in Moore, Tessier‐Lavigne, & Kennedy, 2007; Chisholm *et al.,* 2016; Evans, 2016). In order to respond to these extracellular cues, the subcellular localization of guidance receptors is carefully orchestrated. In *D. melanogaster*, a number of commissural axons cross the midline through the anterior commissure of each body segment in response to a Wingless/Integrated (WNT) repulsive cue (Yoshikawa *et al.,* 2003), and then extend anteriorly on the contralateral side in response to Slit (SLIT) cues (Kidd, Bland, & Goodman, 1999) (Fig. 3). The axon guidance receptor Derailed (DRL), which detects the WNT signal, is enriched in the commissural region of the axon but is absent from the distal part (Callahan *et al.,* 1995; Bonkowsky *et al.,* 1999). Conversely, the Roundabout molecules ROBO2 and ROBO3, which detect SLIT cues, accumulate on the distal part of the axon (past the midline) and are excluded from the commissures in the proximal part of the axon (Kidd *et al.,* 1998a,*b* *;* Katsuki *et al.,* 2009). Thus, these receptors define two adjacent compartments within the axon, a proximal one with DRL, and a distal one with ROBO2 and ROBO3, which reflect the sequential encounters of the axon with the different guidance cues. Based on fluorescence recovery after photobleaching (FRAP) experiments in cultured neurons, Katsuki *et al*. (2009) elegantly demonstrated the existence of a diffusion barrier located at the junction between the proximal and distal regions, which is required for the restricted localization of ROBO2, ROBO3, and DRL. These transmembrane molecules are motile within each compartment but do not cross the compartment boundary (Katsuki *et al.,* 2009). However, remarkably, in low‐density cultures the localization of these receptors also resembles the *in vivo* pattern, indicating that intrinsic properties regulate their axonal compartmentalization even in the absence of extracellular cues (Katsuki *et al.,* 2009) (Fig. 3B). These results reveal that a neuron possesses a cell‐autonomous ability to establish axonal sub‐compartments defined by the specific localization of axonal guidance receptors.

**Figure 4:**
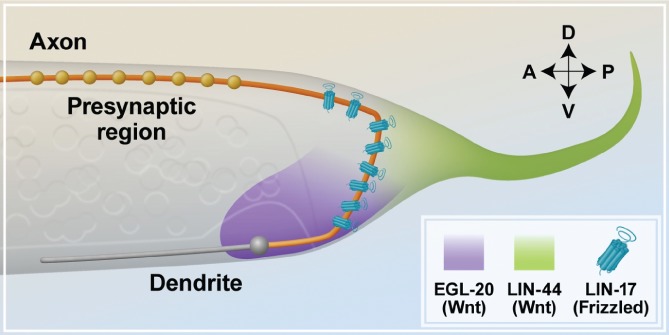
Sub‐compartmentalization of the LIN‐17/Frizzled receptor in *Caenorhabditis elegans*. In the DA9 motor neurons, a posterior gradient of the LIN‐44/Wnt ligand (green) and a ventral gradient of EGL‐20/Wnt (purple) define the axonal sub‐compartmentalization of LIN‐17/Frizzled receptors, which in turn confines the localization of presynaptic RAB‐3 molecules (gold circles) to the distal segment of the DA9 motor neuron axon (orange). EGL, egg‐laying defective; LIN, abnormal cell lineage; Wnt, wingless.

In *C. elegans*, the DA9 cholinergic motor neuron provides another example of axonal compartmentalization. The soma of this neuron is located in the ventral nerve cord at the posterior end of the body. Its axon first runs posteriorly, then circumferentially to reach the dorsal side of the animal, and finally anteriorly to innervate the dorsal muscles; the DA9 dendrite extends anteriorly from the ventrally located soma along the ventral side of the animal (Fig. 4). Previous studies have shown that the axon of the DA9 neuron contains two compartments: a proximal region, which is asynaptic, and a distal region, which contains presynaptic loci (Fig. 4). Interestingly, this compartmentalized axonal organization is regulated by the WNT receptor abnormal cell lineage (LIN‐17/Frizzled), which clusters in the proximal asynaptic region of the axon to inhibit the formation of synaptic loci in this area (Klassen & Shen, 2007). This is a remarkable example of negative regulation of synaptic organization, a process that is typically regulated by active targeting of synaptic proteins to presynaptic boutons (Pinto & Almeida, 2016). The compartmentalized localization of LIN‐17 results from the balanced response of the receptor to the posterior secreted signals of LIN‐44/Wnt and a more anterior and ventral signal of egg‐laying defective EGL‐20/Wnt (Fig. 4). Thus, in this system proximal restriction of LIN‐17 leads to the distal compartmentalization of presynaptic molecules (Klassen & Shen, 2007).

Several examples of dendritic compartmentalization have also been reported. In mouse granule cells (GCs), which are located in the olfactory bulb and are involved in odour discrimination, the β3 subunit of gamma‐aminobutyric acid receptors (GABARs) is clustered in the soma and dendritic shafts but not in the spines (Nunes & Kuner, 2015). These GABAR β3 subunits are required for the correct inhibition of the GCs and the downstream inhibition of mitral cells via dendro‐dendritic synapses (Nunes & Kuner, 2015).

Another interesting example is the electrical compartmentalization within dendritic spines that arises not only from the passive electrical properties of the spine, but also from active mechanisms driven by the distribution of ion channels (Yuste, 2013). The dendritic compartmentalization has been largely studied in rat layer 5 neocortical pyramidal neurons, revealing the mechanisms for the integration of excitatory postsynaptic potentials (EPSPs) in the spines and propagation of EPSPs to the soma to generate an action potential (Williams & Stuart, 2000; Atkinson & Williams, 2009; Harnett *et al.,* 2013; Harnett, Magee, & Williams, 2015). The layer 5 neocortical pyramidal neurons have a large dendritic arborization divided into apical dendrites (in layer 1) and basal dendrites (in layers 5 and 6), which represent two defined electric compartments (Williams & Stuart, 2000; Harnett *et al.,* 2015). When the EPSPs are transmitted from the apical dendrites to the soma, their amplitude decreases dramatically (Williams & Stuart, 2000), and this event is controlled by the hyperpolarization‐activated cyclic nucleotide gated (HCN) channels that compartmentalize in the apical dendrites (Williams & Stuart, 2000; Atkinson & Williams, 2009; Harnett *et al.,* 2015). Moreover, HCN channels colocalize with voltage‐gated potassium (K_v_) channels, which have also been implicated in regulating the electrical properties of these neurons (Harnett *et al.,* 2013). Interestingly, recent work in the human layer 5 neocortical pyramidal neurons revealed that a lower density of sodium channels, due to the larger dendritic surface, results in a greater voltage compartmentalization of their apical dendrites compared to the rat neurons, which can confer a higher level of computational potential (Beaulieu‐Laroche *et al.,* 2018).

## IV. COMPARTMENTALIZATION OF INTRACELLULAR COMPONENTS

In addition to membrane molecules, intracellular components, such as organelles, macromolecules and ions, can also be confined to specific domains of a neurite, in a process that is dynamic and often occurs in response to changes in environmental conditions or neuronal activity.

### (1) Compartmentalized location of organelles

Organelles such as mitochondria (involved in the production of the energy‐carrying molecule ATP and in calcium buffering) and lysosomes (essential for the degradation of metabolic products of the cell) can adopt specific and dynamic localizations within neuronal sub‐compartments that reflect the functional needs of the cell (Fig. 1A). Early electron microscopy analysis revealed that presynaptic terminals have an increased number of mitochondria compared to the rest of the neuron, likely to provide the ATP required for neurotransmitter release (Palay, 1956). However, this does not extend to all presynaptic terminals, as later findings identified mitochondria‐free boutons, which were suggested to use different sources of energy such as glycolysis (Shepherd & Harris, 1998; Chavan *et al.,* 2015). In addition to their role in energy support, mitochondria compartmentalize at synapses to buffer excess calcium, thereby regulating the synaptic vesicle cycle (Montero *et al.,* 2000; Vos, Lauwers, & Verstreken, 2010). During the development of cortical neurons, an increasing number of axonal mitochondria become immobile, with more of them localized at presynaptic sites (Lewis *et al.,* 2016).

The presynaptic compartmentalization of mitochondria has been shown to be regulated by synaptic activity. Kinesin‐1, the motor protein required for anterograde transport, associates with mitochondria through a conserved complex formed by the adaptor trafficking kinesin proteins TRAK1/2 (Milton in *D. melanogaster*), and the outer mitochondrial membrane molecule Miro, which acts as a calcium sensor (Macaskill *et al.,* 2009; Wang & Schwarz, 2009). At active presynaptic terminals, Miro senses the high calcium concentration, and destabilizes the Kinesin‐1 transport machinery, resulting in the immobilization of mitochondria at the synapses. Three models have been proposed to account for how this may occur. In the first model, Miro mediates the detachment of Kinesin‐1 from the microtubule together with the Miro‐TRAK complex (Wang & Schwarz, 2009). In the second model, upon sensing calcium, Miro specifically detaches from Kinesin‐1 (Macaskill *et al.,* 2009). In the third ‘engine‐switch and brake’ model (Chen & Sheng, 2013), the high calcium concentration at active synapses generates a stop signal that switches Kinesin‐1 from the Miro‐TRAK1/2 complex to syntaphilin, an axonal protein that anchors mitochondria to microtubules (Kang *et al.,* 2008; Chen, Gerwin, & Sheng, 2009). To date, the mechanisms of mitochondria re‐mobilization from synapses are yet to be elucidated (Sheng, 2017).

Interestingly, upon synaptic sub‐compartmentalization, mitochondria undergo morphological changes, with synaptic mitochondria (sMito) being smaller than the non‐synaptic forms (nsMito) (Fedorovich, Waseem, & Puchkova, 2017; Devine & Kittler, 2018). Recent work has also shown that sMito have a different proteome from nsMito (Volgyi *et al.,* 2015). The different protein levels, which include lower levels of the superoxide dismutase SOD2 in sMito, are consistent with the higher sensitivity of synapses to oxidative stress (Volgyi *et al.,* 2015). Moreover, the tricarboxylic acid cycle proteins, which are involved in aerobic respiration, are also different, reflecting the higher metabolic rate of sMito (Volgyi *et al.,* 2015). It has also been shown that mitochondria compartmentalize in the distal part of the axon when exposed to Parkinson's disease (PD)‐related stress, such as exposure to rotenone (Arnold *et al.,* 2011). In rat primary neurons, this led to increased replication of mtDNA in those distally localized axonal mitochondria, suggesting a possible mechanism that contributes to the susceptibility of neurons to PD (Van Laar *et al.,* 2018).

Although present in smaller numbers, mitochondria can also be found in dendritic spines. A study conducted on live hippocampal neurons revealed that the number of mitochondria in dendritic spines decreases as development progresses, suggesting a role in spine formation in early development (Li *et al.,* 2004). Repetitive membrane depolarization caused an increase in both the percentage of mitochondria per spine and the number of spines containing mitochondria, indicating that they are also involved in the activity‐dependent plasticity of dendrites (Li *et al.,* 2004).

Another highly dynamic organelle is the lysosome. Under normal conditions, lysosomes localize in the soma and can be trafficked by the kinesin and dynein motor proteins (Hirokawa, 1998; King, 2000; Akhmanova & Hammer, 2010). Serum starvation of cultured HeLa cells leads to the compartmentalization of lysosomes in the perinuclear region (Korolchuk *et al.,* 2011). A similar event is observed in cultured cortical neurons of a mouse model of Gaucher disease, a neurodegenerative condition caused by defective lysosomal storage. In this disorder, lysosome compartmentalization may be an early disease marker as it precedes neuronal death (Zigdon *et al.,* 2017). In addition, the dendritic compartmentalization of lysosomes has recently been observed in cultured hippocampal neurons (Goo *et al.,* 2017). In this study, half of the lysosomes were stationary, whereas the other half moved bidirectionally along the microtubules. Importantly, the same study demonstrated that lysosomes localize at the dendritic spines through an interaction with F‐actin in an activity‐dependent manner, suggesting their potential involvement in protein turnover during synaptic activation and remodelling (Goo *et al.,* 2017).

Recently, a surprising role of presynaptic lysosome‐related vesicles was found in the axons of *D. melanogaster* larvae and mouse hippocampal neurons, where they were involved in axonal transport of presynaptic active‐zone proteins and synaptic vesicle proteins (Vukoja *et al.,* 2018). These results highlight the functional significance for these compartmentalized organelles. Although it is evident that organelles can be found in both axonal and dendritic sub‐compartments, it is not known if and how similar events occur in unipolar neurons.

### (2) Compartmentalization of signal transduction molecules

Cyclic nucleotide monophosphates are important second messengers in signal transduction. In *C. elegans* AWC neurons, a pair of olfactory neurons in the head of the animal, cyclic guanosine monophosphate (cGMP) regulates both olfactory sensation (L'Etoile *et al.,* 2002; de Bono & Maricq, 2005; Bargmann, 2006) and odor adaptation (L'Etoile *et al.,* 2002; Lee *et al.,* 2010). Using a genetically encoded cGMP indicator, cGi500 (Russwurm *et al.,* 2007), Shidara, Hotta, & Oka (2017) revealed that the cGMP signal transiently decreases in the AWC cilia (sheet‐like dendrite endings) and increases in both the dendrite and the soma during exposure to odorants, suggesting compartmentalized cGMP activity in the dendrites of sensory neurons.

A second study in *C. elegans* characterized the *in vivo* formation of a transient metabolic compartment which is used to support neuronal function following an increase in synaptic activity (Jang *et al.,* 2016). This is achieved through a redistribution of glycolytic enzymes required for the production of ATP in the two serotonergic neurosecretory motor neurons (NSMs). The enrichment of the enzymes at synaptic sites is mediated by the cell‐autonomous action of synaptic scaffolding proteins, and is required for the local production of ATP to sustain the synaptic vesicle cycle upon energy demand (Jang *et al.,* 2016).

An important study in rat hippocampal neurons has demonstrated an example of dendritic compartmentalization of calcium/calmodulin‐dependent kinase II (CaMKII) (Lee *et al.,* 2009), a molecule that is involved in long‐term potentiation (LTP) following an elevated increase in calcium concentration mediated by N‐methyl‐D‐aspartate (NMDA) glutamate receptors (reviewed in Penny & Gold, 2018). Using a fluorescence resonance energy transfer (FRET) sensor of CaMKII and two‐photon fluorescence lifetime imaging microscopy, Lee *et al*. (2009) showed that the increased activity of CaMKII precedes the spine enlargement during LTP, and that this activation remains compartmentalized within the activated spine. The mechanism for the dendritic compartmentalization of CaMKII activity resides in the fact that its inactivation properties are faster than its diffusion, thereby restricting its activity in the activated spine without spreading to surrounding spines (Lee *et al.,* 2009).

Overall, these findings provide evidence for the compartmentalization of signalling transduction pathways as a mechanism to generate local and rapid neuronal responses to environmental changes.

### (3) Compartmentalized calcium transients

A large body of literature has revealed that cells can compartmentalize not only proteins but also important inorganic molecules, such as calcium ions. Calcium is the most abundant signal transduction molecule controlling key physiological events, which include gene transcription, exocytosis, and cell death (Clapham, 1995), and is also one of the most important signalling molecules for neuronal development and function (Weissman *et al.,* 2004; Zheng & Poo, 2007). In particular, an increase in calcium concentration in the growth cone is required for proper axonal guidance (Sutherland, Pujic, & Goodhill, 2014). Interestingly, an asymmetric local calcium concentration regulates the turning of the growth cone during navigation (Zheng, 2000). The calcium‐sensing family of Homer proteins, which act at postsynaptic sites to regulate calcium homeostasis during synaptic communication, also regulate the calcium concentration in the growth cone during turning (Tu *et al.,* 1999; Gasperini *et al.,* 2009). More recently, another calcium‐sensing molecule, the stromal interaction molecule 1 (STIM1), was found to localize at the endoplasmic reticulum (ER) to regulate the intracellular level of calcium in response to an extracellular cue during turning of the growth cone (Mitchell *et al.,* 2012). Interestingly, STIM1 clusters in puncta on the turning side, further suggesting a function of local calcium in regulating axonal navigation (Mitchell *et al.,* 2012). Similar to STIM1, Homer proteins are associated with the ER (Sandonà *et al.,* 2003), which is a large organelle with different domains and several cellular functions, including calcium storage (reviewed in Koch, 1990; Friedman & Voeltz, 2011). Interestingly, the ER establishes contacts with the plasma membrane and organelles across the entire length of neurons, and these contacts are involved in the regulation of calcium dynamics (Prakriya & Lewis, 2015), suggesting a role for the ER in regulating calcium dynamics in the growth cones during axon guidance (Gasperini *et al.,* 2017). More insights on the structure of the ER, as well as its interactions with the plasma membranes and organelles, have been recently gained by the elegant work conducted in the mouse brain using ion beam‐scanning electron microscopy (Wu *et al.,* 2017). This study also revealed how different ER compartments distribute throughout the neuron, and that the smooth ER, which is the domain free of ribosomes, extends in the axons and forms cisternae at the synaptic sites (Wu *et al.,* 2017).

Neurons also exhibit additional sophisticated mechanisms of calcium compartmentalization important for complex neuronal functions. For example, detecting image motion is a fundamental function for vision that is regulated by the direction‐selective retinal ganglion cells (Euler, Detwiler, & Denk, 2002; Yonehara *et al.,* 2013). These cells comprise a class of retinal neurons that are activated by a visual stimulus moving in one direction and remain silent when the stimulus moves in the opposite direction (Barlow & Levick, 1965). However, the computation of the directional information does not occur in the direction‐selective retinal ganglion cells themselves but rather takes place in retinal interneurons known as starburst amacrine cells, a type of non‐spiking neurons characterized by a highly symmetrical dendritic morphology (Euler *et al.,* 2002). Two‐photon optical recording of calcium transients in these cells has revealed that different dendritic branches respond to light stimuli in a local and independent fashion, and that the calcium response in these branches depends strongly on the direction of movement of the visual stimulus (Fig. 1D) (Euler *et al.,* 2002). This study demonstrates the presence of dendritic calcium compartmentalization in starburst amacrine cells and its critical function in image motion detection (Euler *et al.,* 2002).

Compartmentalization of calcium in dendritic spines is also crucial during the induction of LTP as well as in synaptic‐plasticity‐related functional and morphological changes (reviewed in Chen & Sabatini, 2012). Indeed, calcium microdomains present in the spines lead to selective and compartmentalized activation of LTP molecules, such as CaMKII discussed in Section IV.2 (Lee *et al.,* 2009).

**Figure 5:**
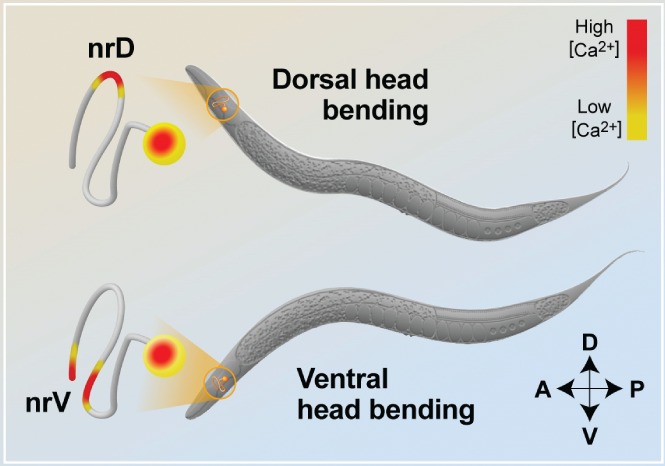
Calcium compartmentalization in the Caenorhabditis elegans ring interneuron A (RIA) axons. During dorsal head bending, calcium transients are detected in the nerve ring dorsal (nrD) region of the RIA axon (see Fig. 2 for details of the RIA neurons), whereas during ventral head bending the calcium transients are recorded in the nerve ring ventral (nrV) regions. These calcium transients are asynchronous and compartmentalized within the axon. The RIA axonal domains also generate synchronous calcium activity upon sensory stimulation.

Compartmentalized calcium activity has also been shown in some interneurons in *C. elegans*. Calcium‐imaging studies, using genetically encoded calcium‐sensitive proteins, have revealed calcium compartmentalization in a pair of head interneurons AIY, with calcium activity occurring in the neurite but not in the soma (Clark *et al.,* 2006; Chalasani *et al.,* 2007). Moreover, the RIA interneurons present asynchronous compartmentalized calcium activity in specific dorsal and ventral regions of the RIA axon, which are located where reciprocal synapses with the dorsal and ventral sensory‐motor SMD neurons are formed (Hendricks *et al.,* 2012) (Fig. 5). The compartmentalization of the calcium transients depends on the cholinergic signalling from the motor neurons, including SMD, and the G‐protein‐linked acetylcholine receptor/muscarinic acetylcholine receptors (GAR‐3/M1/3/5) expressed in RIA neurons. Remarkably, the dorsal and ventral calcium transients are anti‐correlated with each other but respectively correlate with the dorsal and ventral head bending of the animal (Hendricks *et al.,* 2012; Liu *et al.,* 2018). Disrupting the compartmentalized calcium activity in the RIA axon alters the amplitude of head bending, thereby revealing a functional role of calcium compartmentalization in navigational gait. The type of SMD–RIA communication (motor neuron to interneuron) resembles a mechanism known as corollary discharge, in which a copy of a motor command is sent back to the sensory pathway so that the animal can distinguish self‐generated actions from external cues (Crapse & Sommer, 2008a,*b*).

Similar to the role of ER in controlling calcium fluxes in the mammalian growth cone (Gasperini *et al.,* 2017), this organelle could also be responsible for regulating the calcium dynamics in the RIA neurons. In an alternative scenario, mitochondria could be required to shape the calcium dynamics in the axonal compartments of the RIA neurons (Hendricks & Zhang, 2013). In this regard, evidence exists for the mitochondria‐dependent calcium compartmentalization in non‐neuronal tissues, such as the exocrine pancreatic cells, in which calcium gradients regulate fluid secretion (Kasai & Augustine, 1990). Work conducted in the mouse pancreatic acinar cells demonstrated that inositol‐triphosphate‐dependent calcium, which is released from the ER, is compartmentalized in the apical cellular zone by the presence of a ‘mitochondrial belt’ (Tinel *et al.,* 1999). Finally, a third possibility is the presence of calcium‐binding proteins or chelators that could be differentially distributed within axons to buffer calcium, thereby defining the regions of axonal sub‐compartmentalization (Augustine, Santamaria, & Tanaka, 2003; Clapham, 2007). Determining if one or more of these mechanisms are in place is a fascinating and current topic of investigation.

### (4) Neuronal compartmentalization of mRNA and ncRNA

RNAs are transported to different cellular compartments by the microtubule system (Hirokawa, 2006; Lu & Gelfand, 2017). Local messenger RNA (mRNA) translation produces *de novo* proteins in close proximity to their final functional milieu within different cellular compartments. In neurons, both coding RNAs (mRNAs) (Martin & Ephrussi, 2009) as well as non‐coding RNAs (ncRNAs) (Weiss, Antoniou, & Schratt, 2015) can localize at specific subcellular sites to better serve local needs.

#### (a) *Messenger RNAs*

Polysomes, the ribosomal complexes for mRNA translation, are found at the base of dendritic spines of neurons in the dentate gyrus of the rat brain (Steward & Levy, 1982), and relocate into the spines upon stimulation of the neurons (Ostroff *et al.,* 2002). Active ribosomes are also found in the axonal regions and growth cones (Tennyson, 1970; Zelena, 1970; Bunge, 1973; Giuditta *et al.,* 1991). Recently, 2550 mRNAs were identified in the dendrites and the axons of the synaptic neuropil of rat hippocampal neurons, many of which encode known synaptic proteins (Cajigas *et al.,* 2012). Thus, a rich local transcriptome together with the presence of ribosomes suggests the extensive on‐site production of specialized proteins within different compartments of the neurite.

The compartmentalization of specific mRNA to the dendritic spines plays a pivotal role in learning, and is often regulated by the activity of these spines (Frey & Morris, 1997; Redondo & Morris, 2011) (Table 1). A characteristic example is the mRNA of the immediate‐early gene Arc/Arg.3.1 (activity‐regulated cytoskeleton‐associated protein/activity‐regulated gene 3.1), which precisely localizes in the dendritic spines of recently activated synapses (Steward *et al.,* 1998; Dynes & Steward, 2012). Interestingly, local dendritic regulation of mRNA levels of Arc and the 5′AMP‐activated protein kinase (AMPK), by a nonsense‐mediated mRNA decay mechanism, regulates the surface levels of the glutamate receptor GluR1 during synaptic plasticity (Notaras *et al.,* 2018). These findings further reveal diverse mechanisms through which Arc functions as a ‘master regulator’ of synaptic plasticity (Bramham *et al.,* 2010).

**Table 1:** Examples of messenger RNAs (mRNAs) found in dendritic spines.

Gene	Neuronal type/tissue	Reference
Activity‐regulated cytoskeleton‐associated protein (Arc)	Cultured cortical neurons (rat)	Dynes & Steward (2012)
	Dentate gyrus (rat)	Steward *et al*. (1998)
Fragile X mental retardation 1 (FMR1)	Cultured hippocampal neurons (rat)	Antar *et al*. (2004)
		Weiler *et al*. (1997)
Calcium/calmodulin‐dependent kinase II α (CaMKIIα)	Cultured hippocampal neurons (rat)	Shen & Meyer (1999)
Zipcode binding protein 1 (ZBP1)	Cultured hippocampal neurons (rat)	Tiruchinapalli *et al*. (2003)
Spa‐1‐like protein (SPAR)	Cultured hippocampal neurons (rat)	Pak *et al*. (2001)
Postsynaptic density‐95 (PSD‐95)	Hippocampal neurons (rat)	Cho, Hunt, & Kennedy (1992)
Brain‐derived neurotrophic factor (BDNF)	Hypothalamic neurons (mouse)	Liao *et al*. (2012)
Regulator of G‐protein signalling‐7 (RGS7)/Type 5 G protein β (Gβ5) /R7 binding protein (R7BP) complex	Cerebral cortex (mouse)	Aguado *et al*. (2016)

The specific RNA delivery machinery in each active spine has not been fully elucidated. However, the tag‐and‐capture theory of synaptic tagging (Frey & Morris, 1997) might offer a mechanistic view of this delivery system, which relies on the general dendritic transport of mRNAs. Briefly, RNA binding proteins, such as the fragile X mental retardation protein FMRP (Dictenberg *et al.,* 2008), recognize and bind the 3′ untranslated region (UTR), 5′ UTR or coding region of the target mRNAs. The RNA–protein complexes are then transported along the microtubule network in the dendrites by motor proteins. This delivery system has been termed the ‘sushi belt model’ to emphasize the continuous feeding of the local protein factories with mRNAs generated in the cell body (Martin & Ephrussi, 2009; Doyle & Kiebler, 2011).

The presynaptic boutons also have their own local transcriptome composition with important implications for their compartmentalized activity (Akins, Berk‐Rauch, & Fallon, 2009). Similar to the dendritic compartmentalization, the mechanisms of axonal compartmentalization of mRNA molecules are not fully elucidated. Moreover, recent studies have implicated a disrupted on‐site mRNA translation within axons in disease conditions (reviewed in Costa & Willis, 2018). Evidence of mRNAs compartmentalized to presynaptic boutons is summarized in Table 2. A well‐studied example is the neurotransmitter Sensorin of *Aplysia californica* mechanosensory neurons (Schacher *et al.,* 1999; Lyles, Zhao, & Martin, 2006). Synaptic formation of these neurons in culture results in the rapid concentration of Sensorin mRNA at synapses (Lyles *et al.,* 2006), suggesting a role for local Sensorin production in synaptic development. Moreover, during long‐term facilitation (LTF), a form of synaptic plasticity identified in *A. californica*, Sensorin mRNA increases in synaptic regions, resulting in enhanced local protein synthesis (Sun, Wu, & Schacher, 2001; Liu *et al.,* 2003).

**Table 2:** Examples of messenger RNAs (mRNAs) found in presynaptic boutons.

Gene	Neuronal type/tissue	References
Sensorin	Cultured sensory neurons (*Aplysia californica*)	Lyles *et al*. (2006)
		Hu *et al*. (2004)
		Schacher *et al*. (1999)
Cytoplasmic polyadenylation element binding protein (CPEB)	Cultured sensory neurons (*A. californica*)	Si *et al*. (2003)
Fragile X mental retardation protein (FMRP)	Brain (mouse)	Christie *et al*. (2009)

Interestingly, a recent study in *Xenopus laevis* investigated the mRNA trafficking in the axons of retinal ganglion cells (RGCs), with the goal of elucidating the molecular mechanisms for their axonal compartmentalization (Turner‐Bridger *et al.,* 2018). The authors focused on β‐actin mRNA molecules as they are differentially enriched in axons, and their on‐site translation is necessary during branching and turning of the growth cone (Zhang, Singer, & Bassell, 1999; Yao *et al.,* 2006; Donnelly *et al.,* 2013; Wong *et al.,* 2017). Live imaging and single‐molecule microscopy revealed that the majority of the β‐actin mRNAs move by diffusion, and that its axonal compartmentalization in the central region of the growth cone is the result of different speeds between retrograde and anterograde trafficking of mRNAs, during which molecules are individually packaged with ribonucleoproteins (RNPs) (Turner‐Bridger *et al.,* 2018).

#### (b) *MicroRNAs*

MicroRNAs (miRNAs), small non‐coding RNAs which are 21–25 nucleotides in length, play an important role in regulating gene expression. Binding of a miRNA to the 3′ UTR of a target mRNA inhibits translation and/or leads to the degradation of the mRNA (Lee, Feinbaum, & Ambros, 1993; Valencia‐Sanchez *et al.,* 2006). It is estimated that more than 50% of human genes undergo miRNA‐mediated post‐transcriptional regulation (Friedman *et al.,* 2009).

miRNAs regulate the expression of genes at the post‐transcriptional level in a way that can be specific for subcellular compartments, at a great distance from the neuronal cell body. Interestingly, mature miRNAs have been found in both dendrites (Tai & Schuman, 2006; Kye *et al.,* 2007; Bicker *et al.,* 2013) and axons (Wang & Bao, 2017). Moreover, Dicer (a pivotal enzyme in miRNA maturation) and precursor miRNAs (pre‐miRNAs), are present in dendrites and synapses (Lugli *et al.,* 2008; Bicker *et al.,* 2013). Neuronal activity‐evoked calcium signals activate local Dicer (Lugli *et al.,* 2008), which is required for the maturation of a miRNA (miR‐181a) in dendritic spines (Sambandan *et al.,* 2017). Thus, miRNA maturation and function can be executed locally in different neuronal compartments.

Another striking example of compartmental regulation of a miRNA is provided by the brain‐specific miRNA miR‐134 in the rat hippocampal neurons (Schratt *et al.,* 2006). miR‐134 inhibits the translation of mRNA encoding the LIM domain protein kinase Limk1 in the synapto‐dendritic compartment of hippocampal neurons to negatively regulate the size of dendritic spines. Upon stimulation of the neurons, the inhibition of *Limk1* translation by miR‐134 is removed, contributing to synaptic development, maturation and/or plasticity.

#### (c) *Circular RNAs*

Circular RNAs (circRNAs) constitute a divergent class of RNAs which are mainly produced during RNA splicing of protein‐encoding genes. Their common characteristic, the form of a full loop, confers unique properties that have only recently started to emerge (Lasda & Parker, 2014). Interestingly, many circRNAs are expressed in the mammalian brain and are enriched in the synaptoneurosomes (preparations concentrated to obtain the synaptic elements) (Rybak‐Wolf *et al.,* 2015). The puncta of a circRNA named Homer1 are also found in the dendritic processes of cultured hippocampal neurons (You *et al.,* 2015). Together, these findings suggest a potential local role of circRNAs at synaptic sites.

#### (d) *Long non‐coding RNAs*

The long non‐coding RNAs (lncRNAs) are RNA molecules that are longer than 200 nucleotides and do not encode any protein. Many lncRNAs are enriched in the brain tissues and approximately 40% of them are expressed selectively in the mammalian brain (Derrien *et al.,* 2012).

The first lncRNAs studied, the human brain cytoplasmic BC200 and its functional analogue in rodents BC1, localize in the dendritic and axonal domains of the vertebrate neurons and are transported to the dendrites and along the axons (Tiedge *et al.,* 1991; Tiedge, Chen, & Brosius, 1993; Muslimov *et al.,* 1997, 2002). BC1 is also enriched at synapses (Chicurel, Terrian, & Potter, 1993; Lacoux *et al.,* 2012). BC1 and BC200 negatively regulate the initiation of local translation and are critical for the activity of some translation initiation factors (Kondrashov *et al.,* 2005; Lin *et al.,* 2008; Lacoux *et al.,* 2012). These molecules are found specifically in the microdomains of dendritic spines (Kondrashov *et al.,* 2005) and are needed for their structural plasticity. Knocking out BC1 impairs synaptic activity and structural plasticity in the neurons of the mouse barrel cortex and alters social behaviour, implicating BC1 in cognitive functions (Briz *et al.,* 2017).

In conclusion, local protein synthesis in sub‐compartments of neurites not only requires basic translation machinery components, such as ribosomes and mRNAs, but is also regulated by ncRNAs such as miRNAs, circRNAs and lncRNAs. Indeed, recent studies have demonstrated the functional compartmentalization of neurites as a common neuronal property, revealing a highly sophisticated and versatile system of local gene expression that is critical for adaptive neuronal functions.

## V. OUTSTANDING QUESTIONS AND FUTURE DIRECTIONS

Modern neuroscience has made great progress in characterizing functional domains within the morphologically identifiable compartments of neurons. The use of model organisms has facilitated the discovery of the molecular and cellular mechanisms underlying the formation and physiological roles of these sub‐compartments.

However, some of these processes are still not fully elucidated and important questions remain to be addressed. (1) The dendron awaits the identification of the molecular pathways and genetic factors involved in its development and maintenance. (2) For unipolar neurons that contain functionally different synaptic domains, the molecular and cellular events that regulate the specificity of these domains remain to be characterized. For example, only UNC‐101, TTX‐7, and two cyclin‐dependent kinase pathways have been identified as regulators of the polarity of RIA interneurons in *C. elegans*. Other molecules are likely to be required and are not yet discovered. (3) Synaptic mitochondria are crucial for neuronal function and have a specific proteome that differs from that of non‐synaptic mitochondria. What mechanisms underlie the association of different proteins with synaptic versus non‐synaptic mitochondria? (4) Although the compartmentalization of intracellular components has been shown to depend on either the cytoskeleton (as for organelles) or the association with scaffolding proteins (as for the glycolytic enzymes which cluster at the synapse in response to metabolic requirements), the mechanism of calcium compartmentalization in axons and dendritic spines remains largely unknown.

## VI. CONCLUSIONS

(1) The dendritic and axonal compartments of both invertebrate and vertebrate neurons often contain functional domains, which are characterized by distinct molecular distributions, activity patterns, and functional outputs. The formation of these sub‐compartments depends on the integration of intrinsic factors with external cues or on neuronal activity and external stressors.

(2) During early development, axon guidance requires a cell‐autonomous axonal compartmentalization of receptors for guidance cues, as shown in the *D. melanogaster* commissural neurons, and compartmentalization of calcium at the tip of the growth cone to direct turning during navigation towards the target. Moreover, in *C. elegans* neurons the precise clustering of membrane receptors also plays a crucial role in the axonal compartmentalization of synapses.

(3) Once neurons are fully developed, sub‐compartmentalization is still important for neuronal activity and plasticity. For example, compartmentalization of mitochondria at synapses is necessary to supply energy for synaptic transmission, while in the dendrites it is required for the activity‐dependent generation of new spines.

(4) A number of functional compartments contribute to distinct temporal properties of specific neurons. For example, glycolytic enzymes cluster at synaptic loci to support the elevated energy expenditure of high neuronal activity, through an interaction with synaptic scaffolding proteins. Moreover, in the AWC chemosensory neurons of *C. elegans* the level of the second messenger cGMP is locally regulated upon stimulation. The level of cytoplasmic calcium is also locally regulated in the axons of AIY or RIA interneurons of *C. elegans* to encode temporal neuronal activity.
